# CHILDHOOD PHYSICAL ABUSE INCREASES THE RISK OF SUBJECTIVE MEMORY IMPAIRMENT

**DOI:** 10.1093/geroni/igac059.1925

**Published:** 2022-12-20

**Authors:** Lauren Roach, Tyler Bell, Caitlin Pope

**Affiliations:** University of Kentucky, Holland, Michigan, United States; University of California, San Diego, La Jolla, California, United States; University of Kentucky, Lexington, Kentucky, United States

## Abstract

Subjective memory impairment, defined as self-reported difficulties in recall and learning, doubles the risk of Alzheimer’s Disease and related dementia, despite being weakly related to objective memory decline. Because of its strong stability over time, it may be possible that subjective memory impairment reflects earlier life risk factors for dementia such as adverse childhood experiences. It is reported that over a fifth of older adults worldwide experienced physical abuse during childhood. Previous cross-sectional studies suggest physical abuse is associated with later cognitive impairment. Still unclear, are the longitudinal associations between childhood abuse and subjective memory impairment in later life. Using a sample of adults drawn from the Health and Retirement Study (n = 19,185, Mage = 67.05, SD = 11.33) we assessed associations between reported physical abuse by a parent before the age of 18 and subjective memory impairment (current memory problems and perceived memory decline) over periods of up to 18 years. Generalized linear mixed models examined longitudinal associations between childhood physical abuse and subjective memory impairment while controlling for depressive symptoms and other empirically relevant covariates. Experiencing childhood physical abuse was associated with increased likelihood of reporting more current memory problems (OR = 1.17, 95% CI 1.04, 1.33) and perceived memory decline in later life (OR = 1.27, 95% CI 1.13, 1.43). Findings suggest childhood physical abuse is associated with subjective memory impairment, a strong predictor of dementia. Understanding early life conditions, including adverse childhood experiences may help explain associations between subjective memory impairment and dementia risk.

